# Chemical profiling and multidirectional biological effects of the aerial parts of two wild *Commicarpus* species; *C. grandiflorus* and *C. plumbagineus*

**DOI:** 10.1186/s12906-025-05128-x

**Published:** 2025-10-13

**Authors:** Reham Hassan Mekky, Ahmed H. El-Desoky, Riham A. El-Shiekh, Meselhy R. Meselhy, Essam Abdel-Sattar

**Affiliations:** 1 Department of Pharmacognosy, Faculty of Pharmacy, Egyptian Russian University, Cairo-Suez Road, Badr City, Cairo, 11829 Egypt; 2 Pharmacognosy Department, Pharmaceutical and Drug Industries Research Institute, National Research Centre, 33 El Buhouth Street, Dokki, Giza 12622 Egypt; 3 Department of Pharmacognosy, Faculty of Pharmacy, Cairo University, El Kasr El-Aini Street, Cairo, 11562 Egypt

**Keywords:** Commicarpus grandiflorus, Commicarpus plumbagineus, Bioactive agent, Natural enzyme inhibitors, Phenolic composition

## Abstract

**Background:**

*Commicarpus grandiflorus* (A.Rich.) Standl. (CG), and *Commicarpus plumbagineus* (Cav.) Standl. (CP) (Nyctaginaceae family) have long been utilized in traditional medicine for the treatment of various ailments, including urinary tract infections, dysmenorrhea, and blood coagulation disorders.

**Methods:**

The chemical composition of the aerial parts of both plants, total phenolic and flavonoid contents were determined. The antioxidant effects of total methanolic extracts and fractions obtained thereof; methylene chloride (MC) and remaining water (RW) fractions, and their inhibitory potentialson COX-1, COX-2, LOX, ACh, BuCh, and α-glucosidase, were assessed.

**Results:**

The RW fractions of CP and CG exhibited the highest phenolic (57.19 ± 2.43–60.19 ± 1.5 µg GAE/mg) and flavonoid contents (44.32 ± 1.8-53.99 ± 1.8 µg QE/mg), respectively. Also, both fractions demonstrated remarkable antioxidant effects; ABTS^+⋅^ value of 24.85 ± 1.59 and 18.17 ± 1.06 µg/mL, FRAP values of 448.96 ± 37.49 and 773.88 ± 39.13 µMTE/mg, and ORAC values of 9858.06 ± 43.92 and 7666.59 ± 1066.85 µMTE/mg, respectively. Additionally, they exhibited the highest inhibitory against COX-1 (IC_50_ of 1.83 ± 0.05 and 1.86 ± 0.05 µg/mL), COX-2 (0.6 ± 0.1 and 0.69 ± 0.01 µg/mL), LOX (3 ± 0.1 and 2.9 ± 0.1 µg/mL), AChE (11.54 ± 1.34 and 10.52 ± 0.45 µg/mL), BChE (18.63 ± 1.01 and 16.09 ± 0.54 µg/mL), and *α*-glucosidase (86.85 ± 6.66 and 46.69 ± 1.22 µg/mL) enzymes, respectively. A comprehensive analysis identified a total of 42 compounds in both samples, with flavonols, hydroxycinnamic acids, and fatty acids as the predominant classes.

**Conclusions:**

Both samples showed enzyme inhibitory potential against key enzymes linked to chronic diseases. The findings highlight the significant potential of both CG and CP, warranting further research for drug development and biomedicines.

## Background

Noncommunicable diseases (NCDs), often known as chronic diseases, are caused by a variety of physiological, genetic, environmental, and socioeconomic factors. These medical issues develop slowly and last for a long period. According to projections, one-third of the death rate from NCDs can be lowered by 2030 by reducing risk factors such as smoking and alcohol consumption, a lack of proper food, a lack of physical exercise, and providing appropriate disease diagnosis and treatment [1].

Although adjustments in daily lifestyle and nutrition are still required, research indicates that increased dietary antioxidant intake plays an essential role in NCD prevention, particularly when accompanied by a regular diet [2]. Several studies have found that phytochemicals benefit humans, with polyphenols being among the most common and nutritionally relevant phytochemicals. There are currently over 8000 phenolic structures known, with more than 500 occurring in food plants and termed dietary polyphenols [3]. To along with the use of antioxidants, enzyme inhibition is an important strategy in the treatment of NCDs. Despite the fact that enzyme catalysis is essential for life, there are various scenarios in which changes in enzyme activity might result in disease [4]. Infectious illnesses can be treated by targeting specific enzymes of the invading organism that are necessary for survival or reproduction [5]. Changes in genetic and/or environmental variables can cause expression-based, mutation-based, post-translational modification-based, or gain-of-function changes in enzyme activity in chronic disorders [1]. As a result, drugs that block the malfunctioning enzyme may be useful therapeutic treatment options [6].

The Nyctaginaceae family contains around 300 species divided into approximately 30 genera, one of which is *Commicarpus*. A phytochemical analysis of the family revealed the presence of betacyanins, flavonols, phenolics, tannins, saponins, dihydroisofuranoxanthone, rotenoids, and lignans [7]. *Commicarpus grandiflorus* (A.Rich.) Standl. and *Commicarpus plumbagineus* (Cav.) Standl. are traditional medicinal plants found in tropical and subtropical regions around the world (Sudan, Ethiopia, Eritrea, Djibouti, Somalia, Uganda, Kenya, Tanzania, Malawi, Sinai Peninsula, Saudi Arabia, and Yemen). Other synonyms of *C. grandiflorus* include *Boerhavia dichotoma* G.W.Schimp. ex Oliv., *Boerhavia fruticosa* Dalzell, *Boerhavia grandiflora* A.Rich., and *Boerhavia plumbaginea var. grandiflora* (A.Rich.) Asch. & Schweinf. Synonyms for *C. plumbagineus* include *Boerhavia commersonii* Baill., *Commicarpus commersonii* (Baill.) Cavaco, *Commicarpus verticillatus* (Poir.) Standl., *Boerhavia repanda* Roxb., *Boerhavia verticillata* Poir., and *Boerhavia dichotoma* Vahl [7]. Methanolic extracts of the aerial portions of these two *Commicarpus* species growing in Saudi Arabia have been found to demonstrate strong anti-trypanosomal and antiprotozoal activity [8]. The detailed biological activities and chemical profiling of *C. grandiflorus* and *C. plumbagineus* are yet to be investigated. As a result, this study attempts to determine their chemical profiles, antioxidant activities, and enzyme inhibitory abilities against the important enzymes connected to chronic diseases.

## Materials and methods

### Chemicals and instrument

A microplate reader (Tecan, USA) was used. Folin-Ciocalteu reagent, anhydrous aluminum chloride, quercetin, gallic acid, trolox, sodium hydroxide, anhydrous sodium carbonate, fluorescein, 2,2’-azino-bis-3-ethylbenzthiazoline-6-sulphonic acid (ABTS), ferrous tripyridyltriazin, phosphate buffer, acetonitrile, *Saccharomyces cerevisiae* α-glucosidase enzyme, *p*-nitrophenyl-D-glucopyranoside, acarbose, *p*-nitrophenyl butyrate, and orlistat were purchased from Sigma–Aldrich (St. Louis, MO, USA).

### Plant material

In March 2013, aerial parts of *C. grandiflorus* and *C. plumbagineus* were collected along the Al-Hadda Road in Saudi Arabia’s Western Region. The plant was authenticated by Dr Farag A. Al-Ghamdi, Department of Biology, Faculty of Science, King Abdulaziz University, Jeddah, Saudi Arabia. The voucher specimen (CG-1126) was placed at the herbarium of the Department of Natural Products and Alternative Medicine, Faculty of Pharmacy, King Abdulaziz University, Jeddah, Saudi Arabia. Plant materials were air-dried in the shade and then ground.

### Extraction and fractionation

Dried powdered plant parts of *C. grandiflorus* and *C. plumbagineus* (aerial parts, 30 g each) were refluxed with methanol (3 × 100 mL). The solvent was distilled under low pressure, and the methanolic extracts were stored at 4 °C for future biological in vitro experiments. Crude methanolic extracts (3 g) were diluted in water and separated by liquid partitioning with methylene chloride (3 × 50 mL), which was then collected and evaporated to yield MC fractions (0.90, and 0.70 g, respectively). Where the leftover aqueous layers were collected and evaporated to get RW fractions (2.05, and 2.27 g, respectively). All were examined using the relevant in vitro model.

### Total phenolics and flavonoids

The total phenolic contents of the samples were determined using the Folin-Ciocalteu method [9]. The absorbance of the resulting blue colour was measured at 750 nm with a microplate reader (Tecan, USA). Total phenolic contents of the extracts were expressed as *µ*g gallic acid equivalents (GAE) per mg of plant extract. All samples were analyzed in triplicate.

Total flavonoidal content of the samples was determined by the aluminium chloride colourimetric assay [9]. The absorbance of the resulting yellow colour was measured at 415 nm with a microplate reader (Tecan, USA). Total flavonoid contents were expressed as *µ*g quercetin equivalents (QE) per mg of plant extract. All samples were analyzed in triplicates.

### Biological activities

#### Cyclooxygenase 1 (COX-1), cyclooxygenase 2 (COX-2), and lipoxygenase (LOX) inhibition assay

The inhibitory COXs activity [10] was assayed calorimetrically using Cayman colorimetric COX (ovine) inhibitor screening assay kit (Cayman Chemical Company, MI, USA), according to the manufacturer’s instructions. 5-LOX inhibitory assay [11] was carried out colorimetrically using lipoxygenase inhibitor screening assay kit (Cayman Chemical Company, MI., USA) according to the manufacturer’s instructions.

#### α-glucosidase inhibition assay

α-Glucosidase [5]; *p*-nitrophenyl-D-glucopyranoside (5 mM), glucosidase enzyme (1 U/mL in 0.1 M phosphate buffer, pH 6.9), and different concentrations of GSE (0.00781-1 mg/mL) were added and then incubated for 20 min at 37 °C. The absorbance was measured at 405 nm. Acarbose was used as standard. Each experiment was carried out in triplicate.

#### Acetyl and butyryl choline esterase enzymes (AChE, and BuChE) Inhibition assays

The assays were undertaken by the standard technique [12]. Butyrylthiocholine iodide and acetylthiocholine were utilized as substrates in BChE and AChE assays, respectively, where DTNB was served as indicator. All samples were dissolved in MeOH. The intensity of the developed color was measured at 405 nm using a microplate reader.

#### ABTS, FRAP, and ORAC

Antioxidant activities of GSE were assessed using 2,2’-azino-bis-3-ethylbenzthiazoline-6-sulphonic acid (ABTS) [13], Ferric Reducing Antioxidant Power (FRAP) [14], and oxygen radical absorbance capacity (ORAC) [15] antioxidant capacities.

#### LC/MS chemical profiling

##### Sample preparation for LC/MS analysis

One mg of each extract was dissolved in 2 mL of methanol. After membrane filtration (0.45 mm) aliquots of the solutions were subjected to LC/MS analysis. The whole procedure was performed in dim light.

##### LC-PDA/ESI-MS analysis

Liquid chromatography-photodiode array-electrospray ionization–tandem mass spectrometry (LC-PDA/ESI-MS) analysis was performed as previously described [16]. LC-ESI-MS analysis: HPLC (Waters Alliance 2695) and MS spectrometry (Waters 3100). The mobile phase was ready by filtering via membrane disc filter (0.45 μm) after that degassed by sonication. For gradient elution, the mobile phase was solvent A (0.1% formic acid (FA) in H_2_O) and solvent B (0.1% FA in CH_3_CN/MeOH (1:1; *v*/*v*)). The linear gradient profile was as follows: 95% A (5 min), 95–90% A (10 min), 90–50% A (55 min), 50–95% A (65 min), and 95% A (70 min), with an injection volume of 10 µL. The flow rate (0.6 mL/min) was split 1:1 before the MS interface with negative ion mode parameters (source temperature 150 °C, desolvation temperature 350 °C, cone gas flow 50 L/h, cone voltage 50 eV, capillary voltage 3 kV, and desolvation gas flow 600 L/h). Spectra were recorded in the ESI in both negative and positive ionization modes between 50 and 1000 *m/z*. The peaks and spectra were processed using the Maslynx 4.1 software.

##### Molecular networking

The MS/MS (MS2) data in both positive and negative modes were independently uploaded as mzml files to the publicly accessible Global Natural Product Social molecular networking (GNPS) platform (https://gnps.ucsd.edu/ProteoSAFe/status.jsp?task=2435210b1418473eb9855ffb4abe685f, Accession on 17 Aug 2022).

### Statistical analysis

Data are presented as mean ± standard deviation (SD) after they were subjected to one way analysis of variance (ANOVA) followed by (Tukey) post hoc test at level of *P* ≤ 0.05. using statistical analysis system IBM SPSS Statistics 22 (Armonk, NY, USA) [17]. It was also used for Pearson correlation determination. As for conditional formatting and multivariate data analysis, they were performed by Microsoft Excel 365 (Microsoft, Redmond, WA, USA) and XLSTAT v5.03 (Lumivero, Denver, CO, USA) softwares, respectively.

## Results and discussion

### Total phenolics and flavonoids assays

RW of both samples showed the highest phenolic and flavonoids contents which were 57.19 ± 2.43 µg GAE/mg extract, and 44.32 ± 1.8 µg QE/mg extract, for CP and 60.19 ± 1.5 µg GAE/mg extract, and 53.99 ± 1.8 µg QE/mg extract, for CG **(**Table 1**)**. The investigation of phytochemical constituents within the plant family remains relatively uncommon. In the literature, nothing could be tracked regarding the chemical composition of the genus *Commicarpus.* Only a limited number of reports have briefly mentioned the presence of flavonols, flavones, and phenolic compounds [18–20]. The therapeutic properties of medicinal plants are closely linked to their antioxidant capacities. Phenolic compounds have been identified as significant contributors to the antioxidant potential of these plants. Interestingly, numerous studies have indicated that the highest yield of phenolic antioxidants is often obtained from mother liquor extracts rather than pure water extracts [21]. In agreement with our results RW of both plants showed the highest phenolics.

**Table 1:** Total contents and biological activities of the total extracts, methylene chloride, and remaining water fractions of *C. grandiflorus* and *C. plumbagineus*

Sample	CG TE	CG MC	CG RW	CP TE	CP MC	CP RW	Standard
Total phenolics^@^	20.92 ± 0.44	28 ± 2.9	60.19 ± 1.5	24.05 ± 0.46	31.22 ± 0.61	57.19 ± 2.43	-
Total Flavonoids^$^	23.01 ± 0.23	26.11 ± 1.2	53.99 ± 1.8	21.01 ± 0.5	33.81 ± 0.64	44.32 ± 1.8	-
FRAP^#^	302.87 ± 13.95^a^	292.15 ± 15.23^a^	448.96 ± 37.49^b^	510.99 ± 35.46^b^	263.16 ± 22.30^a^	773.88 ± 39.13^c^	-
ABTS*	34.48 ± 0.74^e^	26.47 ± 1.82^d^	24.85 ± 1.59^cd^	9.59 ± 0.56^a^	22.96 ± 1.18^c^	18.17 ± 1.06^b^	5.84 ± 0.23
ORAC^#^	7084.39 ± 191.70^b^	4824.11 ± 175.33^a^	9858.06 ± 43.92^c^	7141.57 ± 10.44^b^	5428.03 ± 82.18^a^	7666.59 ± 1066.85^b^	-
COX-1*	3.93 ± 0.05^c^	3.83 ± 0.05^c^	1.83 ± 0.05^a^	3.96 ± 0.05^c^	2.96 ± 0.05^b^	1.86 ± 0.05^a^	0.6 ± 0.1 Indomethacin
COX-2*	0.83 ± 0.05^cd^	0.96 ± 0.05^d^	0.6 ± 0.1^b^	0.96 ± 0.05^d^	0.3 ± 0.01^a^	0.69 ± 0.01^bc^	0.6 ± 0.02 Indomethacin
LOX*	6 ± 0.1^d^	4.96 ± 0.05^c^	3 ± 0.1^a^	7 ± 0.1^e^	3.86 ± 0.05^b^	2.9 ± 0.1^a^	5.96 ± 0.15 Zileuton
Glucosidase*	466.6 ± 5.7^f^	331.2 ± 1.62^d^	86.85 ± 6.66^b^	373.1 ± 9.06^e^	298.8 ± 2.22^c^	46.69 ± 1.22^a^	116.7 ± 2.14 Acarbose
AChE*	41.57 ± 2.45^c^	24.6 ± 1.23^b^	11.54 ± 1.34^a^	37.23 ± 1.67^c^	28.53 ± 1.9^b^	10.52 ± 0.45^a^	0.25 ± 0.01 Donepezil
BuChE*	78.63 ± 3.0^c^	38.40 ± 1.25^b^	18.63 ± 1.01^a^	89.45 ± 3.05^d^	33.83 ± 1.8^b^	16.09 ± 0.54^a^	0.29 ± 0 Donepezil

^@^The results were expressed as µg Gallic acid equivalent (GAE)/mg extract

^$^The results were expressed as µg quercetin equivalent (QE)/mg extract

^#^µMTE/mg sample

^*^IC_50_ (µg/mL)

There are no significant differences between the same letters in the same column at (*P* ≤ 0.05)

### Biological activities

Age-related diseases or NCDs, such as arthritis, diabetes, dementia, cancer, cardiovascular disease, osteoporosis, Alzheimer’s disease (AD), and metabolic syndromes, are frequently linked to oxidative stress. Reactive oxygen species are naturally formed in the body to regulate cellular functions such as cell survival, stress responses, and inflammation. However, an overabundance of reactive oxygen species can upset the equilibrium between antioxidants and prooxidants, resulting in oxidative stress. Recent studies have highlighted the relevance of natural substances with antioxidant features in lowering oxidative stress and improving immunological function [22].

In the 21 st century, in vitro assays have emerged as critical tools for studying the underlying mechanisms involved in the treatment of Alzheimer’s disease, a degenerative disorder characterized by memory loss and cognitive decline. AD is influenced by a number of complicated variables, including cholinergic toxicity, insulin resistance, neuroinflammation, and oxidative stress. Medicinal plants are rich in antioxidant phenolic chemicals and are used ethnomedical to treat inflammation, diabetes, and cognitive impairment. As a result, they can be multi-target neuroprotective medicines against Alzheimer’s disease that meet the criterion for containing a variety of bioactive ingredients with low toxicity. Ideally, treatments for multifactorial AD should exert many effects in order to successfully halt disease development [23]. The extracts were evaluated for their abilities to inhibit key enzymes associated with chronic diseases, including cyclooxygenase 1 (COX-1), cyclooxygenase 2 (COX-2), lipoxygenase (LOX), acetylcholinesterase (AChE), butyrylcholinesterase (BuChE), and α-glucosidase.

The elevation of the expression and activity of oxidative stress enzymes is evident in AD, which causes biomolecular damage and neuronal death [24]. Plants naturally contain a variety of phytochemicals, including phenols, flavonoids, alkaloids, glycosides, lignins, and tannins. Among these, phenols and flavonoids are the most common phytoconstituents known for antioxidant activity. Flavonoids’ B-rings include hydroxyl groups, which allow them to donate hydrogen atoms during free radical reactions. Furthermore, phenolics are potent antioxidants through several methods, including scavenging free radicals, donating hydrogen atoms, quenching singlet oxygen, chelating metal ions, and acting as substrates for radicals such as superoxide and hydroxyl [25]. In this study, we evaluated the antioxidant potential of the total extracts and fractions of the aerial parts of *Commicarpus grandiflorus* (CG) and *Commicarpus plumbagineus* (CP). The ABTS scavenging properties, FRAP reducing power, and quenching of oxygen radicals’ assay (ORAC) were employed to assess their antioxidant activity. Encouragingly, all samples exhibited promising antioxidant potential. Particularly, the RW fractions, the fractions containing a higher concentration of phenolic compounds, demonstrated the highest antioxidant activities among the samples **(**Table 1**).**

Furthermore, multiple complicated toxicities, such as endoplasmic reticulum stress and telomerase dysfunction, can cause neuroinflammation and the production of inflammatory proteins, aggravating cognitive deficits. Microglial cells serve as an important function in triggering immunological responses and inflammation in the brain. Microglia are activated by oxidative stress, Aβ buildup, and other neurotoxic causes, resulting in the production of cytokines and inflammatory mediators, contributing to acute neuroinflammation. Anti-inflammatory and antioxidant medications have the ability to reduce immune-mediated damage and oxidative stress [24]. In this study, the authors selected 3 inflammatory mediators COX-1, COX-2, and LOX to document the anti-inflammatory activity of the investigated plants. It is worthwhile to highlight the remarkable anti-inflammatory activity of *Commicarpus* species, namely the RW fractions, which could provide a source of naturally occurring bioactive phytoconstituents with potential biological applications **(**Table 1**).**

AChE and BuChE inhibitors have been created as pharmacological medications to treat Alzheimer’s disease, a neurodegenerative condition characterized by memory loss and cognitive decline. The reasoning for suppressing AChE and BuChE is based on the assumption that increasing the availability of acetylcholine at cholinergic receptors in the brain promotes the transmission of impulses between neurons, therefore boosting cognitive function [26]. All samples demonstrated good abilities to inhibit AChE and BuChE, with the same view that RW fractions dominated the potency.

Furthermore, Type 2 diabetes, which is associated with hyperinsulinemia and insulin resistance, can promote neurodegeneration and cognitive impairment in Alzheimer’s patients, as well as impaired brain glucose metabolism. Both conditions share comparable characteristics; medicinal plants that might enhance insulin secretion would assist both diabetics and Alzheimer’s patients [24]. Meta-analyses have found that those with type 2 diabetes are 56% more likely to acquire Alzheimer’s symptoms. Reduced insulin levels and poor insulin receptor sensitivity in the brain can lead to Aβ toxicity and tau buildup. The expression “type 3 diabetes” has been coined to characterize the appearance of diabetes-related symptoms in AD patients [23]. In our study, we the samples exhibited good inhibition against α-glucosidase reflected their role in the multifaceted burdens in AD such as insulin resistance. RW fractions of both plants displayed the highest activity among the tested samples **(**Table 1**).**

### LC/MS chemical profiling and molecular networking

**Fig. 1:**
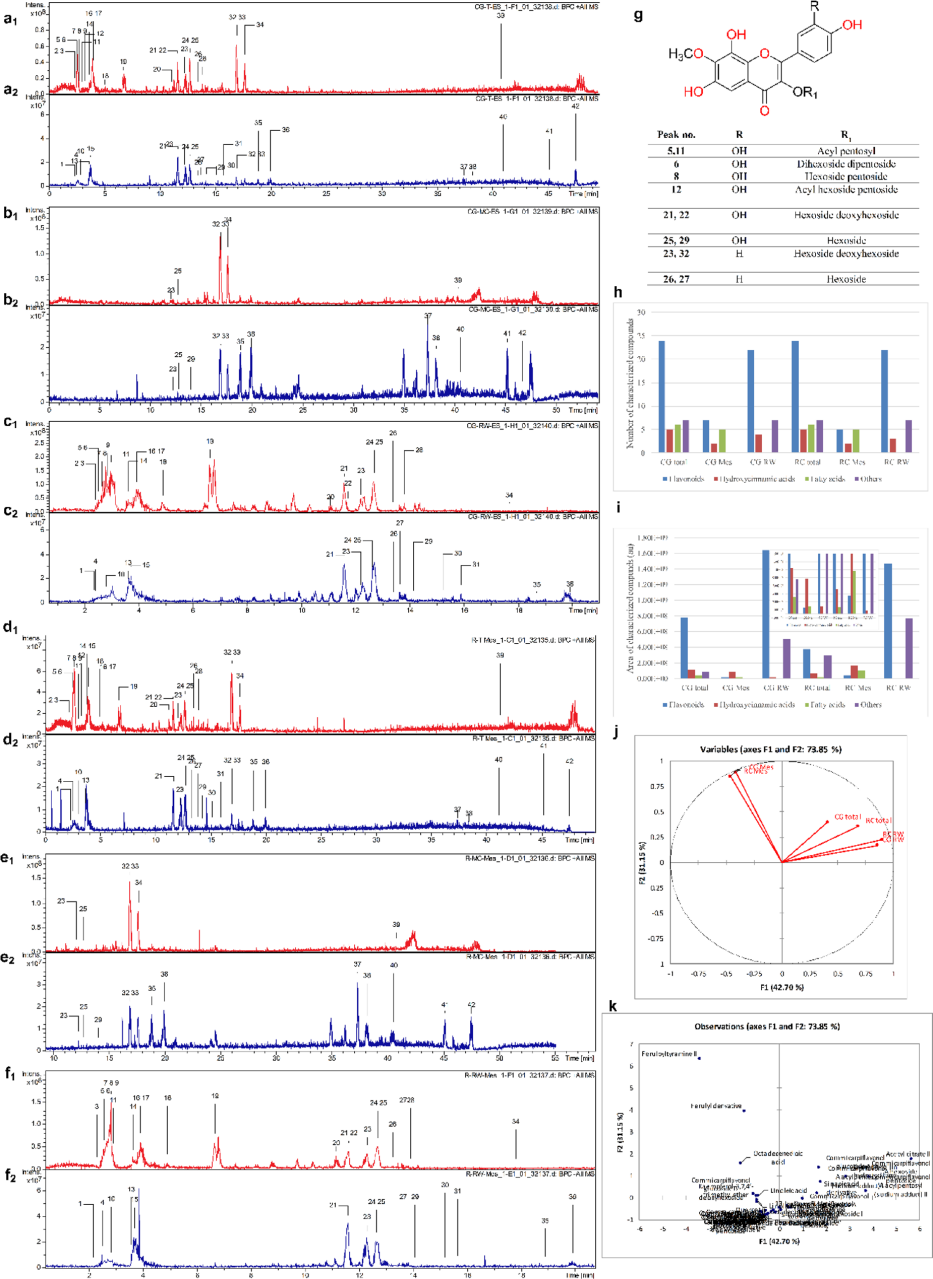
Base peak chromatograms with the numbers of the characterized metabolites in CGTE (**a**), CG MC (**b**), CG RW (**c**), and CP TE (**d**), CP MC (**e**), CP RW (**f**) in the positive (1) and negative (2) ionization modes, Structures of observed Commicarpiflavonols (**g**), Comparison of the characterization results of the studied extracts and fractions (**h**) metabolites number, (**i**) metabolites area, (**j**) PCA score plot, and (**k**) loading plot

The metabolic profiling of the total methanolic extracts (TE) of *C. grandiflorus* (CG) and *C. plumbagineus* (CP) alongside the methylene chloride (MC) and remaining water (RW) fractions in positive and negative modes revealed the presence of 42 metabolites that were basically sub-grouped into phenolics (29) and non-phenolics (13). The strategy of the characterization based on observation of the molecular ion peaks and accompanied neutral losses with the UV absorbance maxima with consulting Reaxys database, PubChem database, Chemspider database, and relevant literature *via* the Egyptian Knowledge Bank [16–29]. The profiles of the two species were quite similar as in Fig. 1a-f which describes the base peak chromatograms of the total extracts, and their respective fractions (methylene chloride and remaining water) which were quite similar and hence it indicates the chemical similarity between the two species.

### Phenolic compounds

The presence of phenolics was widely figured out in the extracts of CG and CP with the presence of six hydroxycinnamic acid derivatives and 24 flavonoids.

**Fig. 2:**
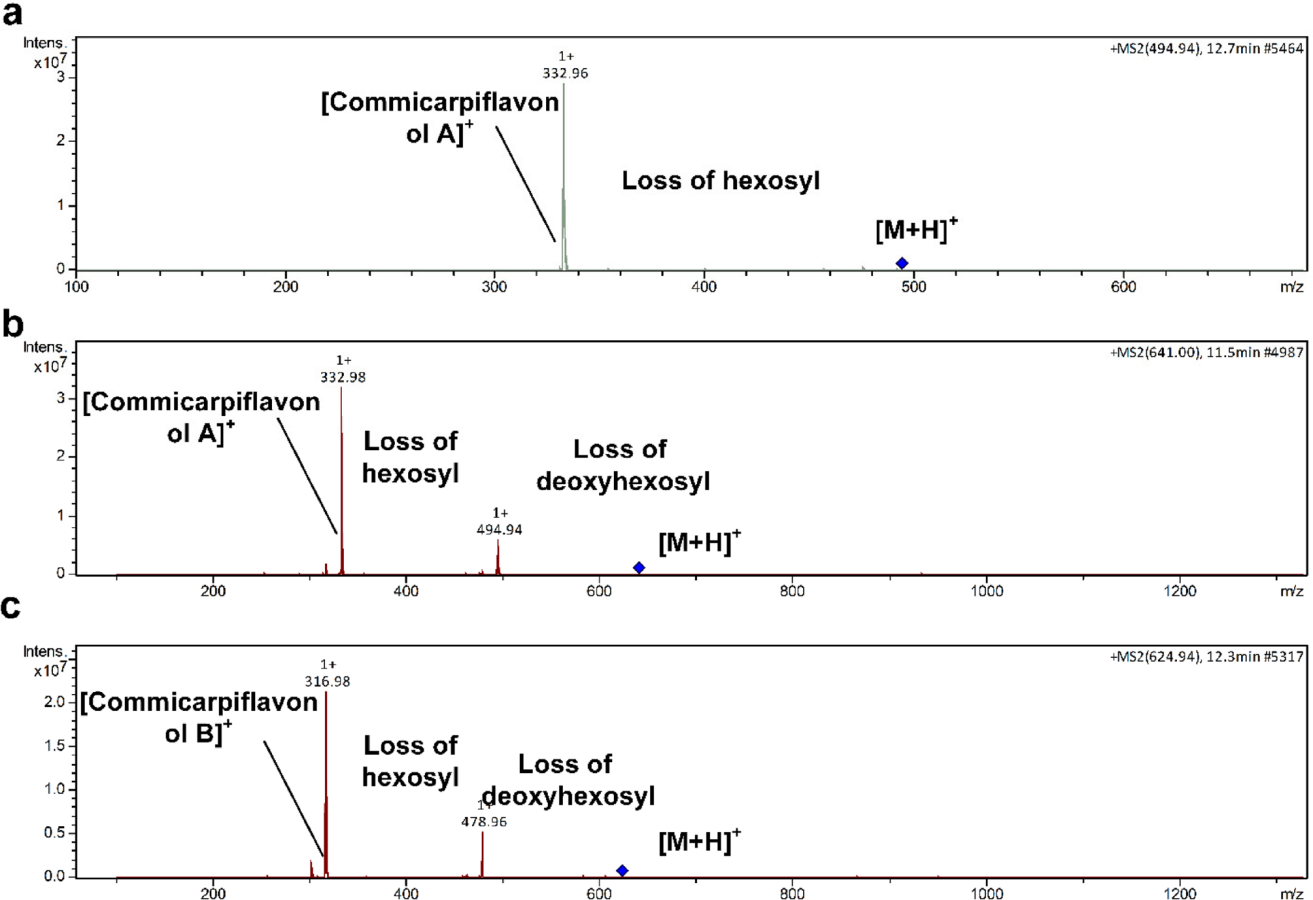
Pattern of fragmentation of commicarpiflavonol A hexoside I (**a**), commicarpiflavonol A hexoside deoxyhexoside I (**b**), and commicarpiflavonol B hexoside deoxyhexoside I (**c**)

**Fig. 3:**
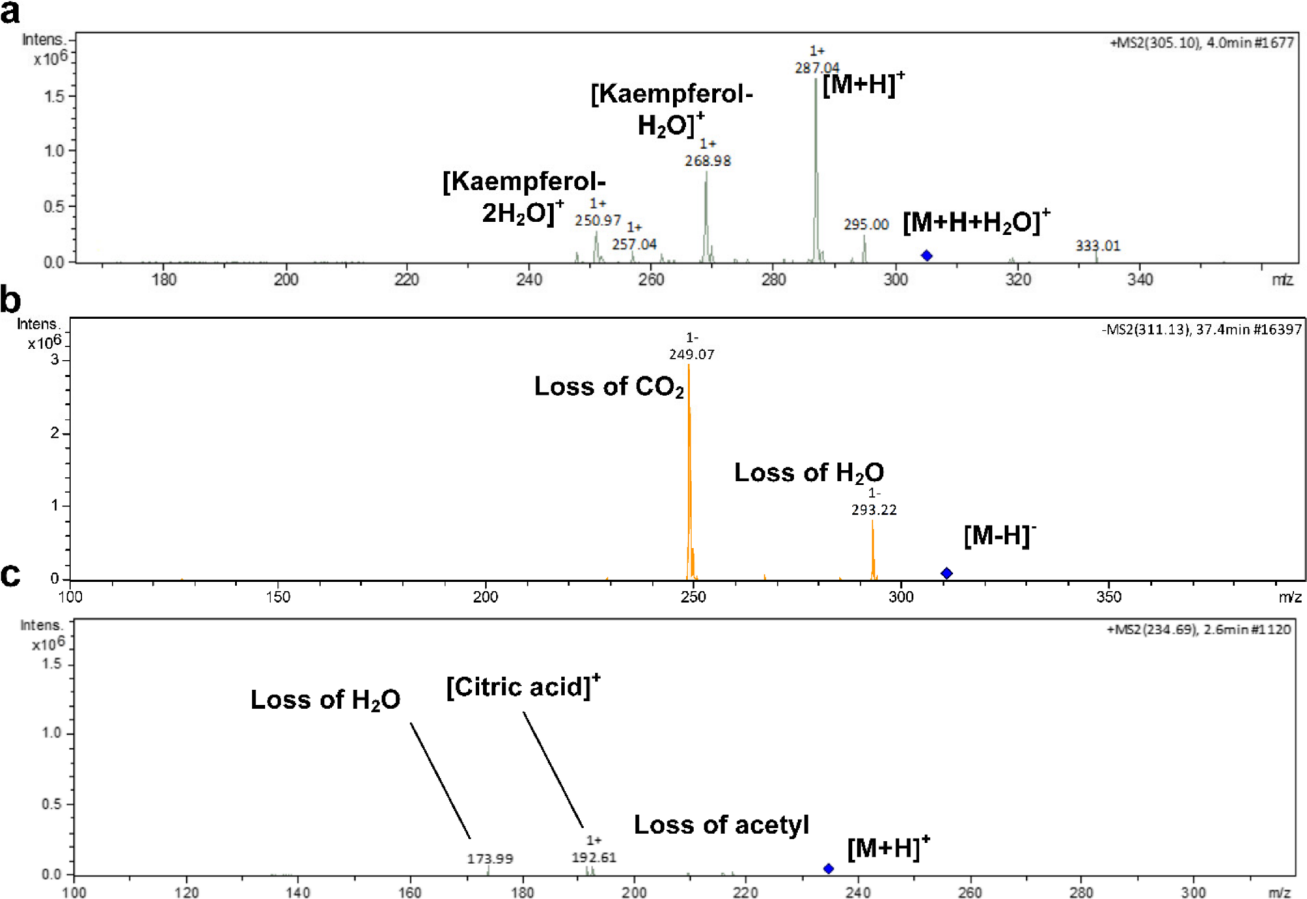
Pattern of fragmentation of kaempferol (in hydrated form) (**a**), Octadecenedioic acid (**b**), and acetyl citrate I (**c**)

With regard to flavonoids, it bears noting that they were classified to flavonols (19), dihydroflavonols (1), flavanols (1), flavan-3-ols (1), and flavones (2). In this sense, the presence of flavonols was mainly as derivatives of commicarpiflavonols A and B [7]. In this sense, peaks 25 and 29 were annotated as two isomers of commucarpiflavonols A hexoside with *m/z* 493 and 495 for [M-H]^−^ and [M + H]^+^, respectively. They exerted the characteristic neutral loss of a hexosyl moiety (−162 Da) with the presence of the aglycone at *m/z* 331 and 333 in the negative and positive ionization modes, respectively (Table 2, Fig. 2a). Similarly, peaks 21 and 22 represented commicarpiflavonols A hexoside deoxyhexoside I-II that were noticed with additional sequential loss of a deoxyhexosyl moiety (Table 2, Fig. 2b). Besides, peaks 5 and 11 were characterized as commicarpiflavonol A acylpentosyl I-II that were observed as an ion with sodium adduct at *m/z* 529 accounting for [M + H + Na]^+^. They expressed the neutral loss of acetyl pentosyl (174 Da). In addition, peaks 8 and 12 accounted for commicarpiflavonol A hexoside pentoside and its acetylated derivative respectively, Table 2. In this context, peak 6 with *m/z* 921 [M + H]^+^ was characterized as commicarpiflavonol A dihexoside dipentoside (Table 2). As per Reaxys database, the presence of commicarpiflavonols A derivatives were not described before and hence they are considered as new proposed structures except for commucarpiflavonols A hexoside I-II. As for commicarpiflavonol B derivatives, they were characterized by the presence of commicarpiflavonol B aglycone (*m/z* 317 [M + H]^+^. In this sense, peaks 26 and 27 were characterized as commicarpiflavonol B hexoside I-II [7], whereas, peaks 23 and 32 accounted for commicarpiflavonol B hexoside deoxyhexoside I-II with additional loss of deoxyhexosyl moieties (Table 2, Fig. 2c) which were mentioned before as setidenosides A in the aerial parts of *Cirsium setidens* Nakai (Asteraceae) [30]. Regarding kaempferol derivatives, they were represented as kaempferol in hydrated form (peak 17, Table 2, Fig. 3a). Meanwhile, peak 35 accounted for kaempferol 3,7,4’-trimethyl ether. In line with quercetin derivatives, they occurred as quercetin hexoside I-II (peaks 16, 24), quercetin 3,4’ dimethyl ether (peak 36), and a derivative of quercetin dimer (peak 20) which showed the ions of quercetin dimer at *m/z* 603 and quercetin itself at *m/z* 303. A dihydroflavonol was observed as an aromadendrine derivative (peak 14) with the presence of aromadendrine ion and its dehydrated ion (*m/z* 289, 271). Peak 28 with *m/z* 509 exhibited the neutral loss of a hexoside with the appearance of *m/z* 347 which was tentatively identified as (epi)catechin tetramethylether hexoside as an example of a flavan-3-ol. Moreover, two isomers of tricin hexoside I-II were observed with *m/z* 491 as examples of flavones. Peak 7 was annotated as naringenin *C* hexoside pentoside with neutral loss of 60 Da indicating *C*-glycosylation [31].

**Table 2:**
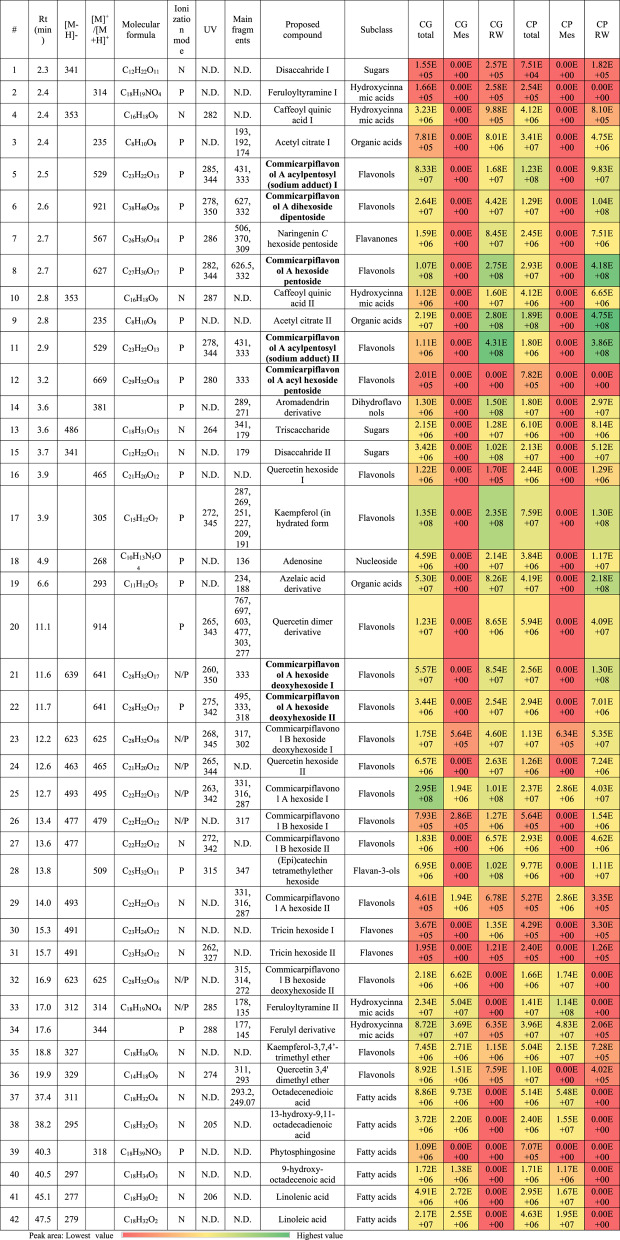
Metabolites characterized in the total extracts (TE), methylene chloride (ME), and remaining water (RW) fractions of *C. grandiflorus *and* C. plumbagineus*

The letter codes I, II, etc. indicate different isomers

Concerning hydroxycinnamic acid derivatives, peaks 4 and 10 were annotated as caffeoyl quinic acid I-II, where three feruloyl derivatives where observed accounting for feruloyltyramine I-II with another feruloyl derivative with the presence of feruloyl ion (*m/z* 178).

As for non-phenolic compounds, they were classified as fatty acids (6), nucleosides (1), organic acids (3), and sugars. In this sense, peak 38 was characterized as octadecenedioic acid which showed dehydration followed by decarboxylation (Table 2; Fig. 3b). Besides, 13-hydroxy-9,11- octadecadienoic acid and 9-hydroxy-octadecenoic acid were present as they were previously reported in Nyctaginaceae. Peaks 41 and 42 were assigned as linolenic acid and linoleic acid. Three sugars were assigned as disaccharide I-II and trisaccharide that exhibited the neutral loss of hexosyl moieties. Moreover, two isomers of acetyl citrate were observed at peaks 3 and 9 with the presence) of citric acid ion (*m/z* 193) and its dehydrated form (Table 2; Fig. 3c). Peak 19 was characterized as a derivative of azelaic acid showing the ion of azelaic acid (*m/z* 189). Adenosine as a nucleoside derivative was noticed at peak 18 and exhibited the neutral loss of a pentosyl moiety. In this sense, the chemical structures of commicarpiflavonols detected in the studied extracts and fractions of *C. grandiflorus* Standl and *C. plumbagineus* Standl were presented in Fig. 1g.

### Comparison of the different extracts

The total number of characterized metabolites was 42 metabolites. It bears noting that the total extracts were the richest with 42 metabolites followed by the aqueous fractions whereas the methylene chloride extracts were the poorest **(**Fig. 1h**)**. Upon calculating the relative abundance of each metabolite, it was clear that the aqueous extracts were the richest in the relative amounts of flavonoids, sugars, organic acids, nucleosides **(**Fig. 1i**)**.

A chemometric analysis was performed to compare the differences on the basis of the relative peak areas of all the characterized metabolites by principle component analysis PCA [32] where, the score plot of the PCA showed that the three first components accounted for 87.48% of the total variance in the data. As a matter of fact, the extraction solvent was the main factor of discrimination among the different extracts were the total hydroalcoholic extracts as well as the remaining aqueous fractions were closely related being in the quadrant and they were segregated from the methylene chloride fractions **(**Fig. 1j**)**. The loading plot revealed the segregation of the different extracts/fractions by several metabolites that contribute to the principal components. For instance, feruloyl tyramine II, feruloyl derivative, commicarpiflavonol A hexoside I, and acetyl citrate II that helped to segregate the methylene chloride fractions from the other samples **(**Fig. 1k**)**.

**Fig. 4:**
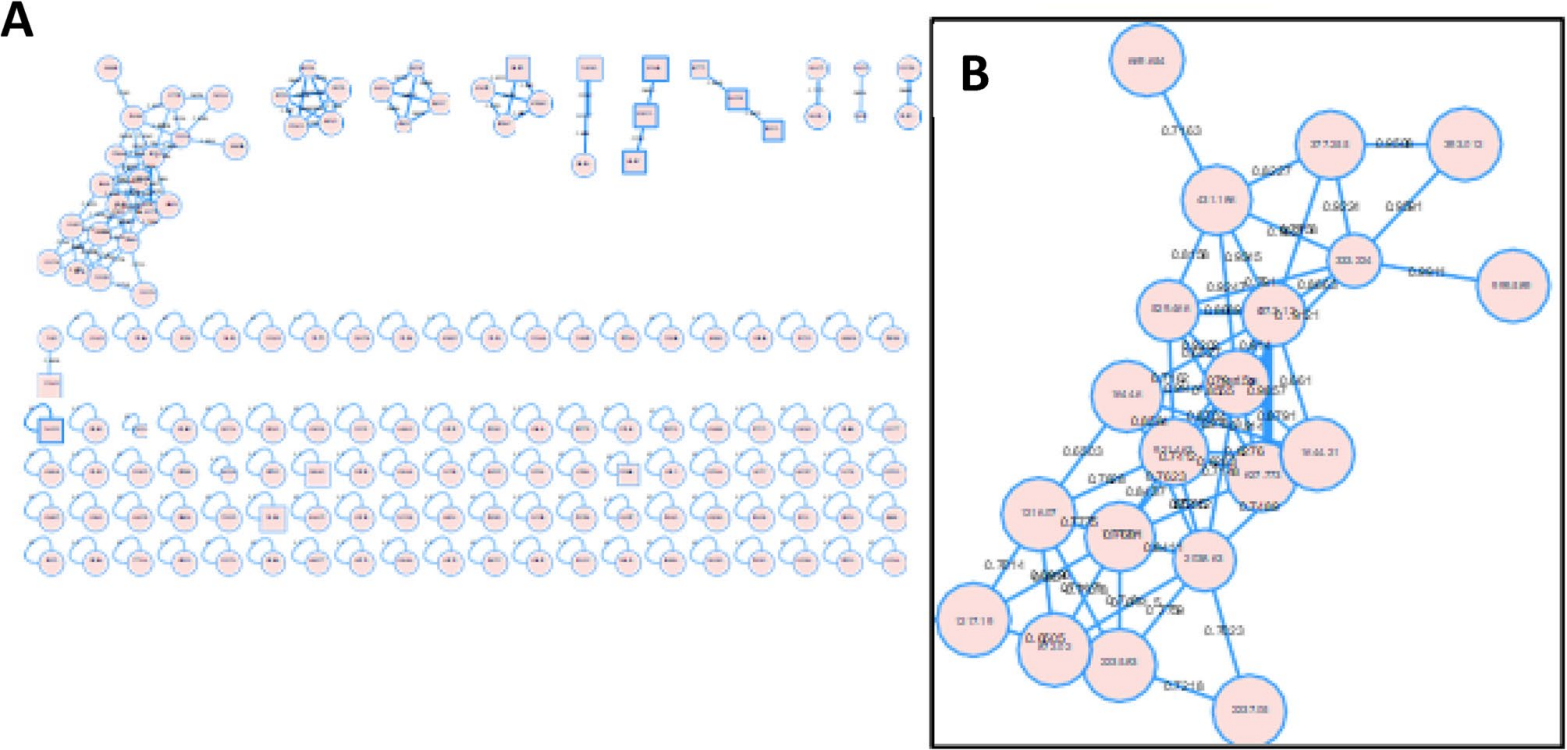
(**a**) Full molecular networking created using MS/MS data in positive mode. The nodes in the enlarged clusters are exhibited as pie charts to reflect the distribution of the ion in the six samples, (**b**) Annotated commicarpiflavonols and their distribution in molecular networking

For the better visualization of the differences and similarities between the investigated samples, molecular networks were constructed using MS/MS data from positive ionization mode. The molecular networks produced 148 nodes divided into 11 clusters (49 connected components, with at least two connected nodes) and 95 singletons **(**Fig. 4A**)**. Annotated clusters within the network are shown in circles belonging to different chemical classes including: Hydroxycinnamic acids, flavonoid glycosides, and fatty acids, while the main class of compounds (commicarpiflavonols) were shown in Fig. 4B. Networks of both plants showed a high degree of similarity as implemented from intersection and difference of both networks as represented in Fig. 4A and B.

### Pearson correlation between TPC, TFC, the relative amounts of bioactive metabolites in CG and CP

Moreover, the relationship between TPC and TFC and the relative amounts of the identified bioactive metabolites viz., flavonoids, hydroxycinnamic acids, and organic acids was stated employing Pearson’s correlation **(**Table 3**).** In this context, a significant strong positive correlation was noticed between TPC and TFC (*r* = 0.965, *p* < 0.01), TPC and relative amounts of flavonoids (*r* = 0. 829, *p* < 0.01), and TFC, and relative amounts of flavonoids (*r* = 0. 775, *p* < 0.01). Moreover, the relative amounts of commicarpiflavonol A/B derivatives exhibited a significant positive correlation with TPC and TFC (*r* = 0.805, *r* = 0.719, *p* < 0.01, respectively) being distinctive markers for CG and CP. Also, a positive correlation was noticed between caffeic acid derivatives and TPC (Table 3).

**Table 3:** Pearson correlation between TPC, TFC, the relative amounts of bioactive metabolites in CG and CP

Correlations							
		TPC	TFC	Flavonoids	Commicarpiflavonol A/B derivatives	Hydroxycinnamic acids	Caffeic acid derivatives
TPC	r	1	0.965^**^	0.829^**^	0.805^**^	− 0.198	0.675^*^
TFC	r	0.965^**^	1	0.775^**^	0.719^**^	− 0.035	0.654^*^
Flavonoids	r	0.829^**^	0.775^**^	1	0.979^**^	− 0.619^*^	0.829^**^
Commicarpiflavonol A/B derivatives	r	0.805^**^	0.719^**^	0.979^**^	1	− 0.630^*^	0.716^**^
Hydroxycinnamic acids	r	− 0.198	− 0.035	− 0.619^*^	− 0.630^*^	1	− 0.594^*^
Caffeic acid derivatives	r	0.675^*^	0.654^*^	0.829^**^	0.716^**^	− 0.594^*^	1

**.Correlation is significant at the 0.01 level (2-tailed)

*.Correlation is significant at the 0.05 level (2-tailed)

Polyphenolics are widely recognized for their diverse range of biological activities, i.e. hydroxy cinnamic acids (compounds 2, 4, 10, 33, 34) such as caffeoylquinic acids, which have been reported to possess antioxidative, neuroprotective, anti-inflammatory, and antidiabetic effects [33]. In our investigated samples, flavonoids emerge as the predominant class of compounds (5–8, 11–14, 16–17, 20–32, and 35–36), offering significant health benefits to humans. Numerous flavonoids, including naringenin and its derivatives [34], aromadendrin [35], kaempferol [36], quercetin and its derivatives [37, 38], catechins [39], and tricin [40], have demonstrated antioxidative, anti-inflammatory, neuroprotective, and antidiabetic activities. However, commicarpiflavonols have not yet been thoroughly investigated for these biological activities. Hence, we recommend isolating and studying these compounds to evaluate their potential effects against enzymes implicated in chronic diseases. Additionally, polyunsaturated fatty acids (compounds 37–42) play a significant role in the prevention and treatment of various pathologies [41]. Their importance in maintaining health and combating diseases cannot be overstated.

## Conclusions

This study sheds light on the biological effects and phytochemical makeup of two important species of *Commicarpus*. The residual water portions of both plants exhibited exceptional antioxidant and metabolic syndrome inhibitory effects, with larger phenolic contents than the other samples. The enzyme inhibitory potentials of the different samples were very comparable. The chemicals found in the highest amounts were quercetin 3,4’ dimethyl ether, a quercetin dimer derivative, quercetin hexoside, commicarpiflavonol A hexoside deoxyhexoside, naringenin C hexoside pentoside, kaempferol-3,7,4’-trimethyl ether, and caffeoyl quinic acid. These chemicals are most likely responsible for the reported biological activity. This study establishes a scientific foundation for the potential use of *Commicarpus* as a source of bioactive compounds for pharmaceutical drug development, which are critical in modulating signaling pathways to improve neurotransmission, neuroplasticity, and cell survival through anti-inflammatory and antioxidant properties. Nonetheless, additional in vitro, in vivo, and clinical research are required, as well as evaluation of the species’ bioavailability and toxicity profile. On the other hand, phytochemical examination using chemometric analysis and untargeted metabolomic networking based on LC-PDS-MS/MS profiles revealed that both investigated *Commicarpus* species are high in flavonoids of the flavonol, dihydroflavonol, flavanol, flavan-3-ol, and flavone kinds. Tandem mass spectrometry revealed the tentative structures of these flavonoids, indicating the presence of five previously unknown flavonol glycosides. Additional phytochemical research is needed to confirm the structures of the identified chemicals and examine their biological functions.

### Study limitations

It is important to interpret our findings in the context of their limitations. The correlation between medicinal plant extracts and multidirectional biological effects seems to be significant; it is crucial to acknowledge that the current body of knowledge primarily relies on in vitro studies. As a result, the extent to which these findings can be applied in a clinical setting remains uncertain. Furthermore, it is important to note that the prediction of bioactive compounds is constrained, particularly when dealing with complex mixtures that involve numerous unknown combination effects. A significant limitation in the utilization of medicinal plants pertains to the absence of standardized extracts. Until recently, the establishment of a clear structure-activity relationship and understanding the mechanisms of action of bioactive compounds have largely been elusive. Gaining further insights into the pharmacology of compounds derived from medicinal plants holds the key to achieving standardized therapeutic regimens. A critical limitation lies in the reproducibility of plant extract compositions, as the properties of the same extract can vary depending on the source. Establishing accurate characterization and authentication methods for bioactive compounds is imperative to implement reliable quality control procedures. Additionally, the availability of high-quality plant species is often restricted to specific geographical regions. Various factors, including plant species and environmental conditions, can significantly impact the availability of reliable sources of medicinal plants.

### Future perspectives

Our study could in the future be followed by in vivo testing in animal models of metabolic syndrome to determine the clinical relevance of such compounds and to establish valid correlation with in vitro results. Additionally, structural modifications of compounds should be explored to enhance their pharmacokinetic and pharmacodynamic properties, along with conducting structure-activity relationship analyses. It is essential to investigate the synergistic interactions within medicinal plant extracts, as well as between compounds and synthetic drugs, to uncover the underlying mechanisms behind their potential activities and identify multiple pathways for targeted interventions. However, it is crucial to note that the interactions between medicinal plant extracts and commercial drugs can exhibit either favorable outcomes, such as synergism, or adverse effects, such as antagonism. Therefore, further research, particularly in vivo studies, toxicity assessments, and investigations into the biomedical potential of these products, is necessary for their recognition as viable biomedical agents.

## Data Availability

The authors declare that the data supporting the findings of this study are available within the paper.
